# The involvement of Neuregulin-1 in the process of facial nerve injury repair through the utilization of dental pulp stem cells

**DOI:** 10.1186/s12903-024-03953-z

**Published:** 2024-02-14

**Authors:** Lihong Yao, Wanqiu Xu, Lixue Liu, Xiaohang Xu, Hualei Xi, Bing Xue, Xiaofang Cao, Song Lin, Guiyan Piao, Jian Sun, Xiumei Wang

**Affiliations:** 1 Department of Stomatology, The Second Affiliated Hospital of Harbin Medical University, Harbin, Heilongjiang Province 150001 China; 2 Mudanjiang Medical University, Mudanjiang, Heilongjiang Province China

**Keywords:** Dental pulp stem cells, Neuregulin-1, Proliferation, Migration, Neural differentiation, Facial nerve injury

## Abstract

**Background:**

Facial nerve injury often results in poor prognosis due to the challenging process of nerve regeneration. Neuregulin-1, a human calmodulin, is under investigation in this study for its impact on the reparative capabilities of Dental Pulp Stem Cells (DPSCs) in facial nerve injury.

**Methods:**

Lentivirus was used to transfect and construct Neuregulin-1 overexpressed DPSCs. Various techniques assessed the effects of Neuregulin-1: osteogenic induction, lipid induction, Reverse Transcription Polymerase Chain Reaction, Western Blot, Cell Counting Kit-8 assay, wound healing, immunofluorescence, Phalloidin staining, nerve stem action potential, Hematoxylin-eosin staining, transmission electron microscopy, and immunohistochemistry.

**Results:**

Neuregulin-1 effectively enhanced the proliferation, migration, and cytoskeletal rearrangement of DPSCs, while simultaneously suppressing the expression of Ras homolog gene family member A (RhoA) and Microfilament actin (F-actin). These changes facilitated the neural differentiation of DPSCs. Additionally, in vivo experiments showed that Neuregulin-1 expedited the restoration of action potential in the facial nerve trunk, increased the thickness of the myelin sheath, and stimulated axon regeneration.

**Conclusion:**

Neuregulin-1 has the capability to facilitate the repair of facial nerve injuries by promoting the regenerative capacity of DPSCs. Thus, Neuregulin-1 is a significant potential gene in the reparative processes of nerve damage.

**Supplementary Information:**

The online version contains supplementary material available at 10.1186/s12903-024-03953-z.

## Introduction

Nerve degeneration or damage can lead to substantial complications and disability [1, 2]. Facial nerve injury, a common traumatic disease in clinical practice, is categorized into five types, with the most severe being the complete rupture of the facial nerve trunk. Current primary clinical treatments include autologous nerve transplantation [3], allogeneic nerve transplantation [4], cell promoting therapy [5], and tissue engineering [6]. Tissue engineering, in particular, shows promise in effectively addressing tissue loss and facilitating morphological recovery.

Seed cells, crucial in tissue engineering, are used to create new tissue or organs for medical purposes. Compared to other Mesenchymal Stem Cells (MSCs) like Bone Mesenchymal Stem Cell (BMSC), Dental Pulp Stem Cells (DPSCs) originate from the neural crest. They are easily accessible, exhibit low immunogenicity, are relatively inexpensive to isolate and culture, have the capacity for multidirectional differentiation, and can be induced into neuron-like cells by various growth factors such as Epidermal Growth Factor (EGF), basic Fibroblast Growth Factor (bFGF), Brain-Derived Neurotrophic Factor (BDNF), Neurotrophin-3 (NT3), Glial cell line-Derived Neurotrophic Factor (GDNF), HLA-27 (B27), and neurobasal mediators [7, 8] Thus, DPSCs hold considerable therapeutic potential for neurological disorders and the advancement of tissue engineering in injury repair. They are derived through the stimulation of various factors and, in the context of nerve injury, have the ability to inhibit neuronal apoptosis, suppress the expression of axon growth inhibitors, and differentiate into neuronal cells [9]. Evidence suggests that DPSCs promote the regeneration of spinal cord axons [10, 11], facial nerve [12], and sciatic nerve [13] in rats with injuries. However, the lack of identifiable DPSCs indicators, along with traditional two-dimensional culture hindering multidirectional differentiation, presents obstacles in the application of DPSCs in tissue engineering. Consequently, exploring target genes to enhance DPSCs function is imperative.

Neuregulin-1 (NRG-1), also known as calmodulin in human biology, is a growth factor controlled by four genes (NRG-1-4), with a sequence similar to EGF [14]. NRG-1 signaling operates through a network including three tyrosine protein kinase receptors: ErbB2, ErbB3, and ErbB4 [15]. This growth factor plays key roles in nervous system development, such as neural differentiation [16], nerve guidance [17], synapse formation [18], myelin sheath formation, and the establishment of the Neuromuscular Junction (NMJ) [19]. However, NRG-1’s impact on pulp stem cells in facial nerve injury restoration is not well-understood.

Although both NRG-1 and DPSCs contribute to nerve regeneration, DPSCs’ effect on nerve regeneration is not yet optimal, and NRG-1 primarily acts on the central nervous system. Studies on the peripheral nervous system are limited, with few related reports. Facial nerve injury is a type of peripheral nerve injury, and it has been reported that NRG-1 subtypes are present in intact and regenerated adult rat nerves. Furthermore, while NRG-1 can regulate the proliferation and migration of periodontal stem cells and other cells, its effect on DPSCs is unreported. Therefore, this study aimed to generate NRG-1 overexpressed DPSCs via lentiviral transfection and to investigate NRG-1’s effect on DPSCs’ regenerative potential in repairing facial nerve injury, clarifying NRG-1’s function in DPSCs and its role in peripheral nerve injury.

## Materials and methods

### Isolation and culture of DPSCs

#### Ethical statements

DPSCs were obtained from male Sprague-Dawley rats weighing approximately 200 g. The rats were sourced from the Laboratory Animal Center of the Second Affiliated Hospital of Harbin Medical University. The animals were housed in a centralized animal care facility with a 12-hour light and dark cycle, and were provided with ample food and water. The experimental protocols involving these cells were duly authorized by the Committee for the Protection and Use of Animals of Harbin Medical University, under Approval No. YJSDW2022-016.

### Isolation and characterization of DPSCs

Mandibular incisors from SD rats were immersed in phosphate-buffered solution (PBS) (Biosharp, Beijing, China) containing 1% double-antibody. Pulp tissues were obtained and digested in trypsin + 3 mg/ml type I collagenase for 50 min. The resulting mixture was centrifuged and added to a specialized medium for stem cells in T25 flasks. The flasks were incubated at 37 °C with 5% CO_2_ for 2–4 generations before being used for subsequent experiments.

### Osteogenic and lipogenic differentiation of DPSCs

The DPSCs were evenly distributed in a 6-well plate (Corning) at an appropriate number. Once the cell fusion reached 70%, the culture medium was replaced with an induced osteogenic differentiation medium (Ori Cell, Guangzhou, China). The medium was subsequently changed every 3 days. After 4 weeks of induction, the cells were fixed with 4% paraformaldehyde for 30 min at room temperature and then treated with an alizarin red working solution. The formation of mineralized nodules of DPSCs was recorded by photographing under a microscope (Nikon).

DPSCs were cultured in 6-well plates(Corning) at a cell density of 2 × 10^4^ cells/cm^2^. Once cell fusion reached 100%, the complete culture medium was replaced with solution A of a lipogenic differentiation medium(Ori Cell, Guangzhou, China). After 3 days of culture, the medium was changed to solution B of a lipogenic differentiation induction medium. Following 24 h of culture, the medium was switched back to solution A. This cyclic culture process was repeated for a total of 21 days. Finally, the cells were subjected to droplet staining with alizarin red O working solution for 30 min. The stained cells were subsequently observed under a microscope(Nikon) and recorded—the formation of lipid droplets.

### Transfection

The cell suspension of DPSCs was prepared and subsequently inoculated into 6-well plates(Corning). After a 24-hour incubation period, the appropriate amount of lentivirus containing NRG-1 was introduced. The cells were then divided into two groups: DPSCs infected with lentivirus lacking the target gene, referred to as the control group (CTRL), and DPSCs infected with lentivirus containing the target gene of NRG-1, referred to as the overexpression group (OV). Reverse transcription polymerase chain reaction (RT-PCR) was performed 48 h after infection to evaluate the efficiency of transfection. Subsequently, the cells were subjected to puromycin screening at a concentration of 2 µg/mL for 2 weeks, resulting in the establishment of stable cell lines.

### Wound healing test

DPSCs were cultured at an optimal cell density. Subsequently, the cell suspension was introduced into a pre-prepared 6-well plate (Corning). Upon achieving complete confluence, a 10 µl pipette tip was placed perpendicular to the 6-well plate (Corning), and the bottom surface was delicately scraped to initiate cell migration. The migratory behavior of cells in each experimental group was recorded through image capture and microscopic observation at 0 h, 24 h, and 48 h after the scratching procedure.

### Cell proliferation assay

Cell suspensions were prepared at a concentration of 5 × 10^4^ cells/ml and subsequently inoculated into 96-well plates (Corning) with 5 replicate wells for each sample. A volume of 100 µl of cell suspension was added to each well, followed by incubation at 37 °C with 5% CO_2_ for 24 h. Subsequently, 10 µl of Cell Counting Kit-8 (CCK-8) working solution (basin, China) was added, and the plates were incubated for 4 hours. The absorbance at 450 nm for 1 day, 2 days, and 3 days was measured using an enzyme labeling instrument (Thermo Scientific) to determine the respective absorbance values.

### Phalloidin staining

The cells were subjected to incubation in a constant temperature incubator set at 37℃ with 5% CO_2_ for a duration of 24 h. After the incubation period, the cells were fixed using a 4% paraformaldehyde solution and treated with phosphate-buffered saline with Tween-20 (PBS-T) to enhance cell permeability. Subsequently, the cells were sealed with PBS with bovine serum albumin (PBS-B) and subjected to staining using Phalloidin staining solution (Beyotime, Shanghai, China) and DIPA (Beyotime, Shanghai, China) in order to visualize the nuclei of the cells. The resulting cytoskeletal staining was then observed, photographed, and documented using a fluorescence-inverted microscope manufactured(Nikon).

### RT-PCR

DPSCs were inoculated at a density of 1 × 10^5^ cells per well in 6-well plates (Corning). Once the cell density reached a high level, total cellular RNA was extracted using Trizol (Takara, Kusatsu, Japan), and the resulting mRNA was reverse-transcribed into cDNA using the Roche Reverse-Transcription Kit. A primer SYBR, cDNA, and ddH_2_O mixture were prepared in specific ratios and subjected to three RT-PCR reactions on a Roche 480 II Real-Time PCR system (Roche Basel, Switzerland). The primer sequences used are provided in Table 1. The CT values were normalized to GAPDH and calculated using the 2ΔΔCt method.

**Table 1:** RT-PCR primer sequences

Gene	Forward primer(5’-3’)	Reverse primer(5’-3’)
NRG-1	CTTCGGTCAGAACGGAGCAA	ACAGTCGTGGAGTGATGGGC
RhoA	AATGTGCCCATCATCCTAGTT	TGTTTGCCATATCTCTGCCTT
F-actin	AGCATTCCAGGACGAATGGG	GAGTGGTGGAATCACCGAGG
S-100	AGGAATGAAGGGCCACTGAGATGT	TTTAATACTTGCAATGCGGTTTTT
GFAP	ACATCGAGATCGCCACCTAC	ACATCACATCCTTGTGCTCC
β3-tubulin	TGCCCCCAGCTTACCTTCCTA	CCCCCTCCAAACACAACC
NSE	TCGCCACATTGCTCAAC	TAACTCAGAGGCAGCCACATC
GAPDH	AACTCCCATTCTTCCACCTTT	CTCTTGCTCTCAGTATCCTTG

### Western blot

DPSCs were inoculated into a 6-well plate (Corning) at a density of 1 × 10^5^ cells per well. Once the fusion reached 100%, the cells were harvested and subjected to treatment with protein lysate (Beyotime, Shanghai, China) and PMFS (Beyotime, Shanghai, China). The protein concentration was determined using the BCA kit (Beyotime, Shanghai, China), and the protein was denatured by steaming at 100 °C for 8 min. An equal amount of total protein was loaded onto the prepared gel plate, and the membrane was sealed with 5% skimmed milk powder after transfer. The primary antibody was incubated overnight at 4 °C with agitation(Table 2). Subsequently, it was combined with the secondary antibody to visualize the immunoblotting bands. A marker (Epizyme, Shanghai, China) can be utilized as a reference for determining the molecular weight position of the target protein.

**Table 2:** Antibody list

Antibody	Manufacturers	Dilution rate
NRG-1	Santa	1:5000
F-actin	Proteintech	1:50000
β3-tubulin	Proteintech	1:50000
NSE	Proteintech	1:50000
NF-κB	Proteintech	1:50000
P38 MAPK	Proteintech	1:50000
GAPDH	Proteintech	1:50000

### Induced neural differentiation

DPSCs were inoculated at a density of 1 × 10^5^ cells per well in 6-well plates (Corning). The induced neural differentiation medium, consisting of 20ng/ml EGF, 20ng/ml bFGF, and 10ug/ml heparin, was added. After 7 days of induction, the cells were divided into two groups: the Ne-CTRL group and the Ne-OV group.

### Immunofluorescence(IF)

After inducing differentiation using the aforementioned method for a duration of 7 days, the cells were subsequently fixed with a 4% paraformaldehyde (PFA) solution and treated with PBS-T to augment cell permeability. Subsequently, PBS-B was applied to seal the cells, and a primary antibody was introduced and left overnight at a temperature of 4℃. On the following day, the primary antibody was combined with a secondary antibody. DAPI-stained nuclei were infected and subsequently observed and photographed using an inverted fluorescence microscope (β3-tubulin, Proteintech, 1:200, NSE, Proteintech, 1:200).

### Construction of animal facial nerve injury model

Male Sprague-Dawley rats weighing between 180 and 200 g were obtained from the Experimental Animal Center of the Second Hospital of Harbin Medical University. The experimental protocol was approved by the Animal Protection and Use Committee of Harbin Medical University (YJSDW2022-016). The rats were housed in a centralized animal care facility, with a 12-hour light-dark cycle and ad libitum access to food and water. General anesthesia was induced by intraperitoneal injection of sodium pentobarbital at a dose of 50 mg/kg. Following anesthesia, the buccal branch of the facial nerve was specifically exposed. A 2 mm truncation procedure was conducted on the facial nerve, subsequently followed by suturing of the truncated end. The tunica albuginea suture was securely closed, and a cell suspension containing 2 × 10^7^ cells, prepared in a volume of 200 µl, was subsequently administered via the tail vein. The rats were randomly allocated into four groups: Control, consisting of rats without facial nerve injury; DPSCs, involving rats with a 2 mm truncation injury to the facial nerve and subsequent tail vein injection of DPSCs; DPSCs-vector, including rats with a 2 mm truncation injury to the facial nerve and subsequent tail vein injection of DPSCs along with an empty vector; and DPSCs-NRG-1, comprising rats with a facial nerve truncation injury and subsequent tail vein injection of DPSCs along with NRG-1. Each group consisted of five rats that were cultured for 2, 4, and 8 weeks.

### Facial nerve trunk action potential detection

Electrophysiologic compound muscle action potentials (CMAP) were recorded using the ADI Powerlab 4/35 electrophysiologic system, which was equipped with a signal filter, in Colorado Springs, CO. The CMAP measurements were performed at 2, 4, and 8 weeks, ensuring the blinding of the CAMP recording. The stimulation mode was set to pulsed mode, with a stimulus intensity of 0.5 mA, wave width of 0.2ms, and a delay of 5ms.

### Transmission Electron microscope(TEM)

The facial nerve tissues were initially treated with glutaraldehyde for fixation, followed by a process of dehydration and embedding. Subsequently, the tissues were appropriately sectioned, and the nerve sheath’s thickness was assessed using a transmission electron microscope. A photographic record was obtained for documentation purposes.

### Hematoxylin-eosin staining(H&E staining)

Facial nerve tissues were procured and subsequently immersed in a 4% PFA solution for a duration of 24 h. Following the fixation process, the tissues underwent dehydration using an ethanol gradient and were subsequently embedded in paraffin. Subsequently, the individual sections of the tissues were subjected to H&E staining. The ensuing morphological alterations were then meticulously observed and documented under a microscope (Nikon).

### Immunohistochemistry(IHC)

The facial nerve of rats was obtained and subjected to a series of procedures, including fixation, dehydration, embedding, sectioning, baking of the film, dewaxing with water, repair using an autoclave repair solution, blocking with a blocking agent, and subsequent closure. The primary antibody was applied overnight at a temperature of 4℃, followed by rewarming for 1 h on the second day. The samples were then combined with the secondary antibody, subjected to DAB chromatography, and stained with hematoxylin to visualize the cell nuclei. The sections were carefully examined and photographed using a microscope (Nikon), and the observations were duly recorded (S-100, Proteintech, 1:1600, β3-tubulin, Proteintech, 1:2400).

.

### Statistical analysis

The results were reported as the mean and standard deviation of three or more independent trials. The data were analyzed using GraphPad Prism software (version 6, MacKiev Software, Boston, MA, USA). The means were compared using a one-way analysis of variance (ANOVA), and t-tests were used to compare the two groups. A *p*-value less than 0.05 was considered statistically significant.

## Results

### Isolation and characterization of DPSCs

**Fig. 1:**
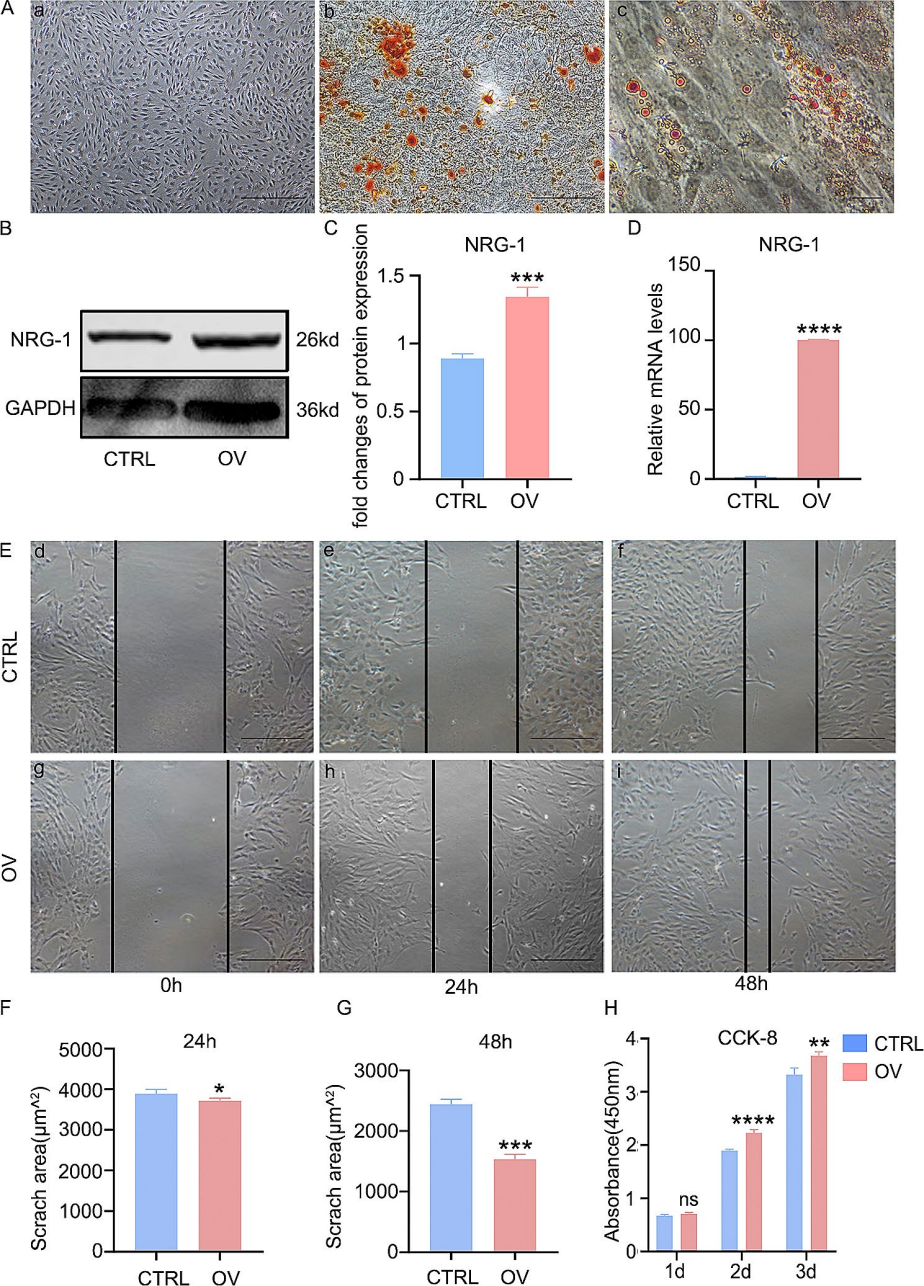
NRG-1 promotes proliferation and migration of DPSCs. (**A**(a)) DPSCs were observed to exhibit a long fusiform and swirling arrangement under the microscope. (**A**(b)) Osteogenic differentiation induced the formation of mineralization nodules. (**A**(c)) Lipid differentiation resulted in the formation of lipid droplets. (**B**, **C**) The transfection efficiency of NRG-1 was assessed through Western Blot detection and subsequent statistical analysis. (**D**) The transfection efficiency of NRG-1 was assessed using RT-PCR. (**E**) The impact of NRG-1 on the migratory capacity of DPSCs at 0, 24, and 48 h was evaluated using the scratch method. (**F**, **G**) The width of the scratch was subjected to statistical analysis. (**H**) The proliferation ability of DPSCs was examined at 1, 3, and 5 days using the CCK-8 method, in order to determine the effect of NRG-1. Statistical significance was determined using the following notation: (ns (*P* > 0.05), ** (*P* < 0.001), *** (*P* < 0.0001), **** (*P* < 0.00001)). The blots in B were cropped

The characterization of DPSCs is currently based on evaluating their morphology, osteogenic, and lipogenic differentiation potential, as distinct markers are yet to be identified. Microscopic examination of primary DPSCs showed a unique elongated, spiral-like arrangement (Fig. 1A (a)). Following osteogenic and lipogenic differentiation medium administration, there was a significant formation of mineralized nodules and lipid droplets, respectively (Fig. 1A(b, c)).

### NRG-1 overexpression promotes the migration of DPSCs

The impact of NRG-1 on DPSCs’ biological behavior was assessed using lentivirus transfection. RT-PCR and Western Blot analyses confirmed a substantial increase in NRG-1 expression in the OV group compared to the CTRL group (Fig. 1B, C, D), indicating successful construction of the NRG-1 overexpression cell model. The scratch experiment revealed a marked increase in the migratory capacity of the OV group, most pronounced 24 h post-scratching (*p* < 0.00001). However, the reduction in scratch width after 48 h was less effective than at 24 h (*p* < 0.0001) (Fig. 1E, F, G).

### NRG-1 overexpression promotes the proliferation of DPSCs

An investigation into NRG-1’s effect on DPSC proliferation was conducted. CCK-8 experiments showed a significant increase in DPSC proliferation in the OV group under NRG-1 exposure, especially on the second day. On the first day, there was no statistically significant difference in cell proliferation between the two groups (Fig. 1H).

### Ability of NRG-1 to promote neural differentiation in DPSCs

**Fig. 2:**
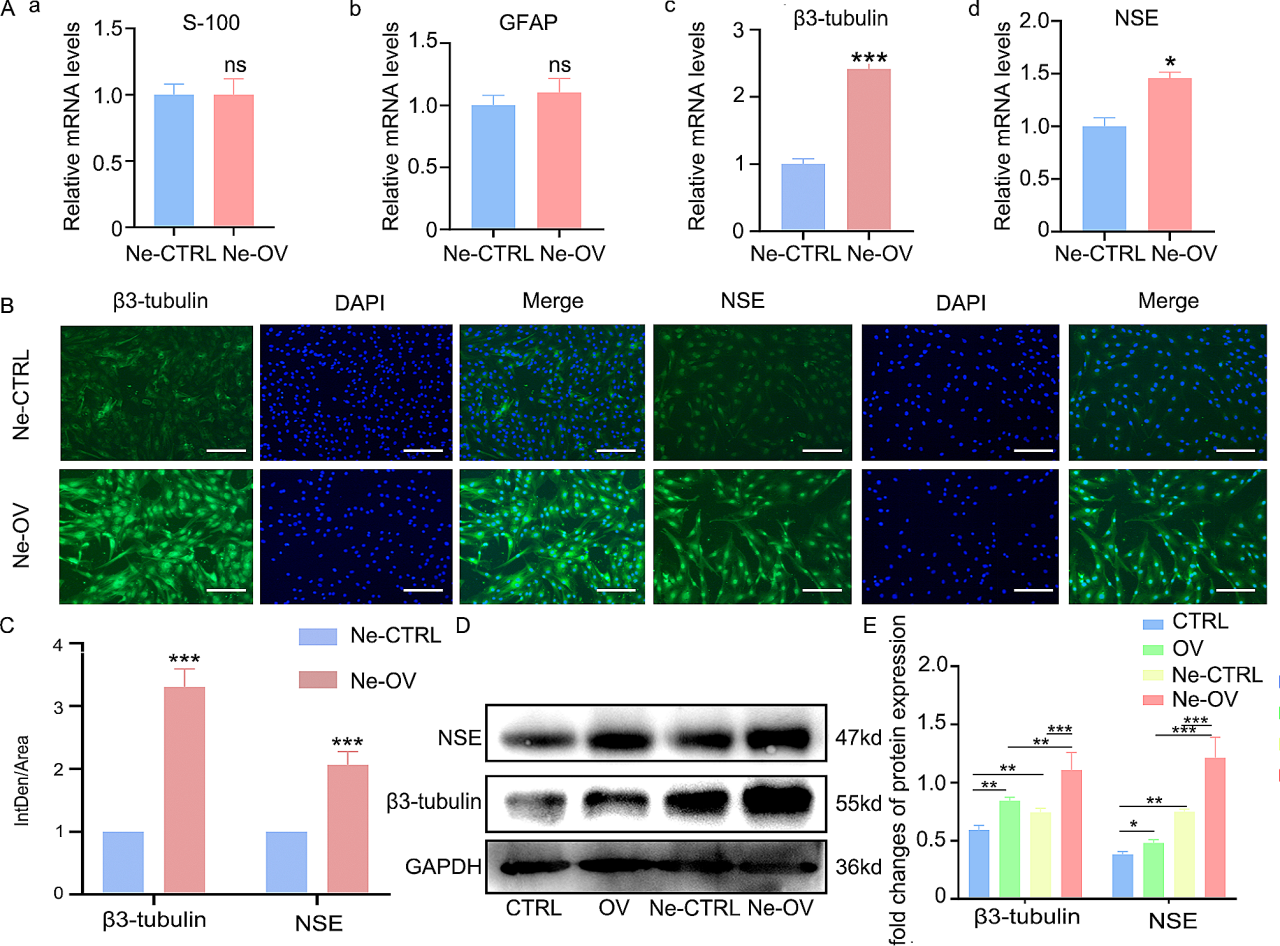
NRG-1 promotes differentiation of DPSCs to neuron-like cells. (**A**) The mRNA expression levels of astrocyte markers S-100 and GFAP, as well as neuronal markers β3-tubulin and NSE, were assessed using RT-PCR. (**B**, **C**) The expression level of the neuronal marker β3-tubulin and NSE was determined through IF and subjected to statistical analysis. (**D**, **E**) The protein expression levels of neuronal markers β3-tubulin and NSE were examined via Western Blot and subjected to statistical analysis. Statistical significance was determined using the following notation: (ns (*P* > 0.05), * (*P* < 0.05), ** (*P* < 0.001),*** (*P* < 0.0001)). The blots in D were cropped

The impact of NRG-1 on the neurotropic differentiation capacity of DPSCs was also investigated. For this purpose, in vitro neurotropic differentiation induction in DPSCs was performed. After a 7-day neural differentiation protocol, RT-PCR analysis showed no significant change in the expression levels of glial cell markers calcium-binding protein (S-100) and Glial Fibrillary acidic protein (GFAP) between the Ne-CTRL and Ne-OV groups (*p* > 0.05) (Fig. 2A(a, b)). However, the Ne-OV group exhibited significantly higher mRNA expression levels of neuron markers β3-tubulin and Neuron Specific Enolase (NSE) compared to the Ne-CTRL group (*p* < 0.0001, *p* < 0.05) (Fig. 2A(c, d)). IF results showed increased fluorescence intensity of β3-tubulin and NSE in the Ne-OV group (Fig. 2B, C). Western Blot analysis also revealed an upregulation in β3-tubulin and NSE protein expression, indicating neuron differentiation, in the Ne-OV group (Fig. 2D, E). These findings suggest NRG-1’s role in promoting DPSC differentiation into neuron-like cells.

### NRG-1 regulates the cytoskeleton

**Fig. 3:**
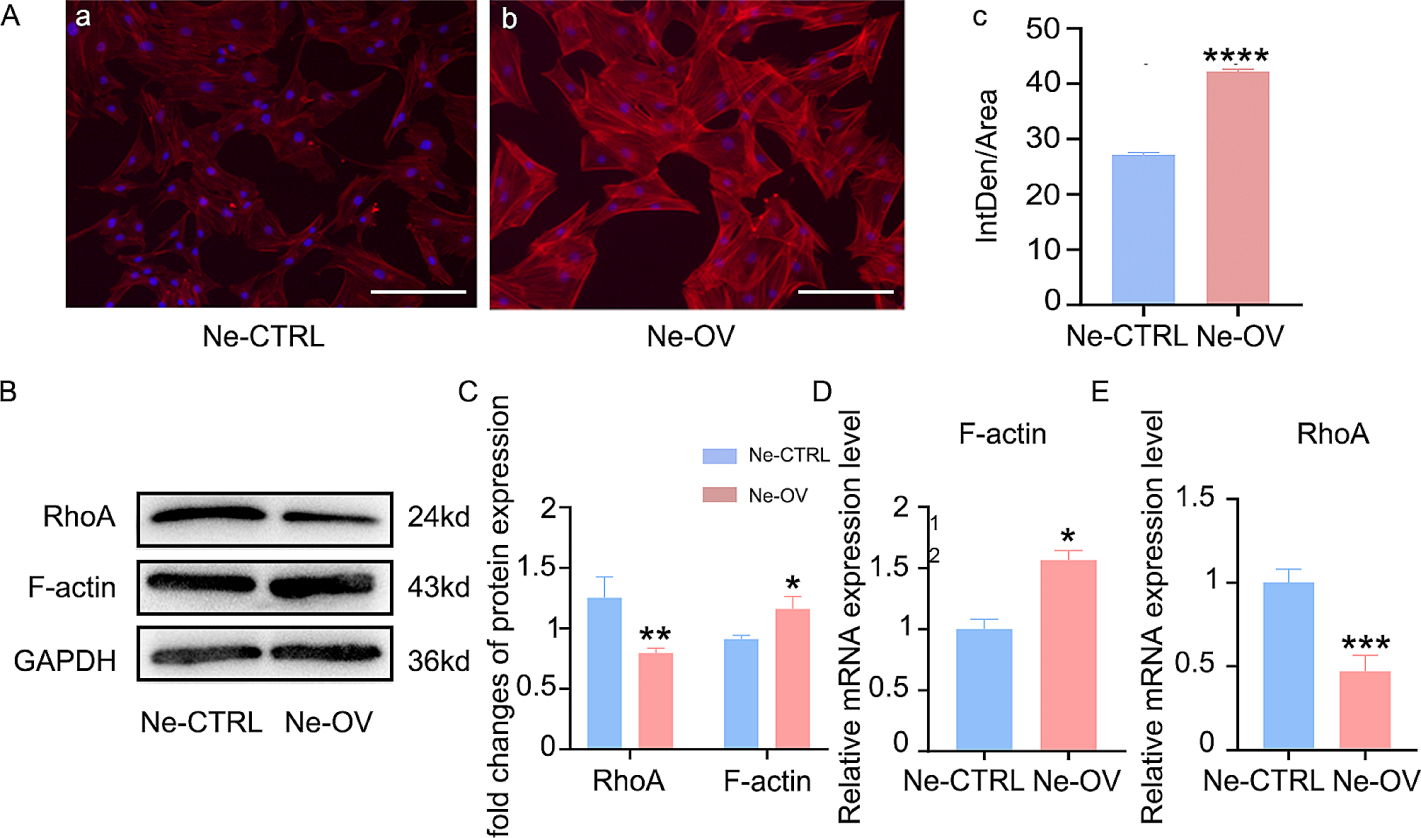
NRG-1 downregulates RhoA expression. (**A**) The cytoskeleton was visualized through Phalloidin staining, followed by statistical analysis. (**B**, **C**) F-actin, RhoA expression was assessed through Western Blot analysis, accompanied by statistical analysis. (**D**, **E**) The expression of F-actin mRNA and RhoA mRNA was detected using RT-PCR. Statistical significance was determined using the following notation: (* (*P* < 0.05), ** (*P* < 0.001),*** (*P* < 0.0001), **** (*P* < 0.00001)). The blots in B were cropped

Further exploration into the mechanism by which NRG-1 facilitates DPSC differentiation into neuron-like cells focused on cytoskeletal changes. We observed alterations in Ras homologous gene family member A (RhoA) in DPSCs with elevated NRG-1 expression. The Ne-OV group displayed increased cytoskeleton fluorescence intensity, cytoskeleton unfolding, and thickening of stress myofilaments (Fig. 3A(a, b, c)). Elevated mRNA and protein expression levels of cytoskeletal actin fibers (F-actin), restrain mRNA and protein expression levels of RhoA were found in the Ne-OV group (Fig. 3B, C, D). RT-PCR analysis showed down-regulated RhoA mRNA expression in the Ne-OV group compared to the Ne-CTRL group (Fig. 3E). At the same time, we found that NRG-1 down-regulated the expression of P38 MAPK and NF-κB by Western Blot (Supplementary Fig. 1). These results indicate that NRG-1 might promote F-actin polymerization by inhibiting RhoA, further enhancing DPSC differentiation into neuron-like cells.

### NRG-1 promotes action potential recovery and axonal regeneration after facial nerve injury

**Fig. 4:**
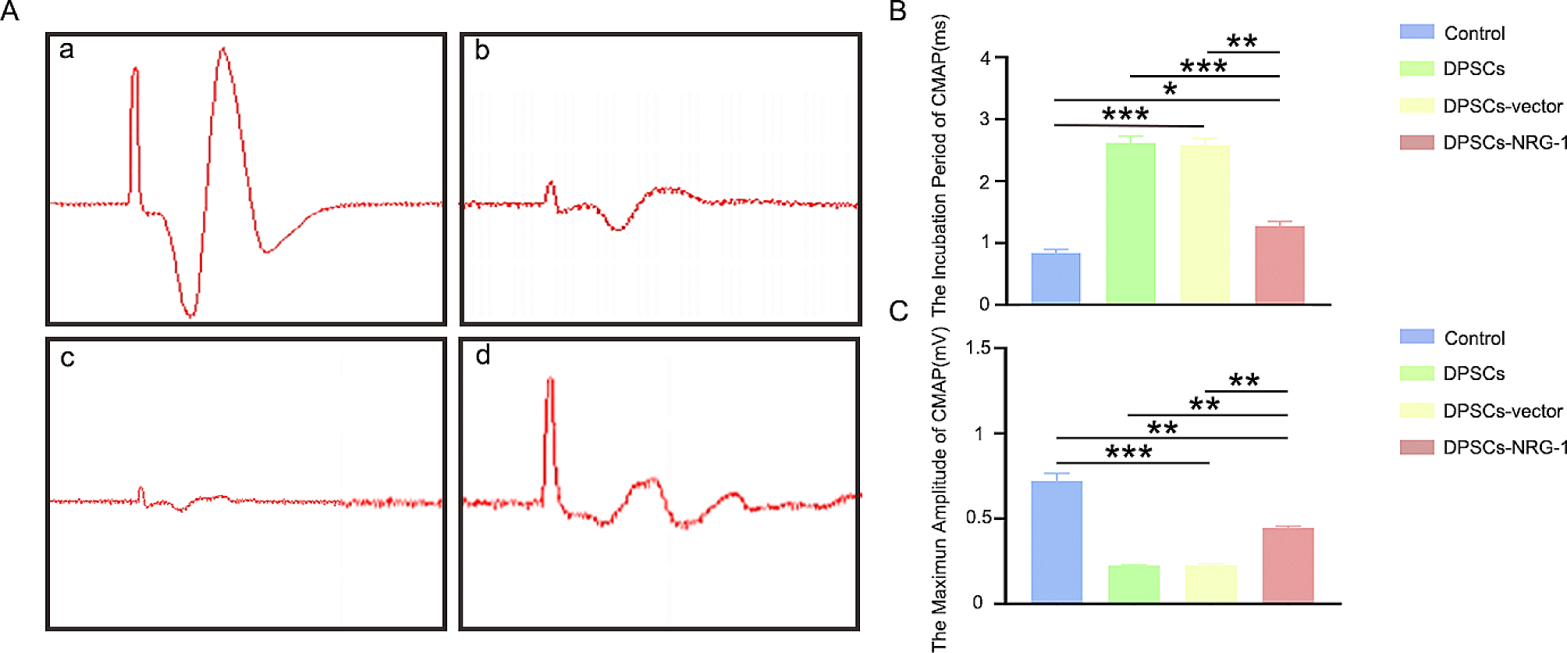
NRG-1 promotes the recovery of nerve trunk action potentials after facial nerve injury in rats. (**A**) The waveform of the action potential in the facial nerve trunk, a: Control, b: DPSCs, c: DPSCs-vector, d: DPSCs-NRG-1. (**B**) Statistical analysis of incubation period. (**C**) Statistical analysis of amplitude. (* (*P* < 0.05), ** (*P* < 0.001), *** (*P* < 0.0001))

**Fig. 5:**
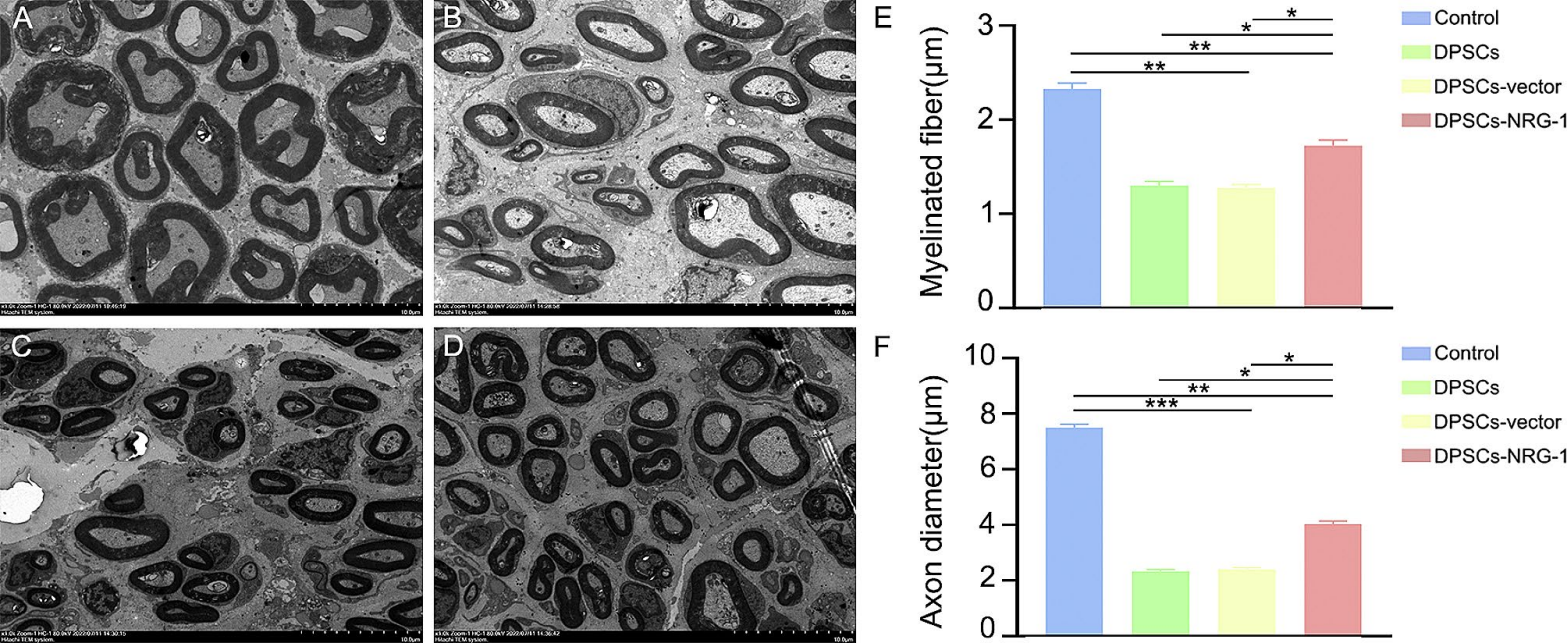
NRG-1 promotes increased myelin thickness and axonal regeneration in the facial nerve. (**A**) The alterations in myelin thickness subsequent to facial nerve injury were examined utilizing transmission electron microscopy. The scale is 10 μm. (**B**) Statistical analysis was conducted to assess the myelinated fiber and axon diameter. (* (*P* < 0.05), ** (*P* < 0.001), *** (*P* < 0.0001))

The study evaluated action potential restoration following facial nerve truncation injury at 2, 4, and 8 weeks. Initially, at 2 weeks, no significant differences in action potentials between groups were noted, with stimulus amplitude present but reflex amplitude absent. By 4 weeks, amplitude increased in all groups, with the DPSCs-NRG-1 group showing the most significant change, yet reflex amplitude was still absent (Supplementary Fig. 2). Notably, at 8 weeks, a continued increase in amplitude was observed in both groups, with reflex amplitude visually confirmed in the DPSCs-NRG-1 group (Fig. 4A). This group also showed improvements in latency and amplitude (Fig. 4B, C). Additionally, TEM histological analysis showed increased axon and myelinated fiber diameter in the facial nerves of rats in the DPSCs-NRG-1 group (Fig. 5).

### NRG-1 promotes repair of facial nerve injury

**Fig. 6:**
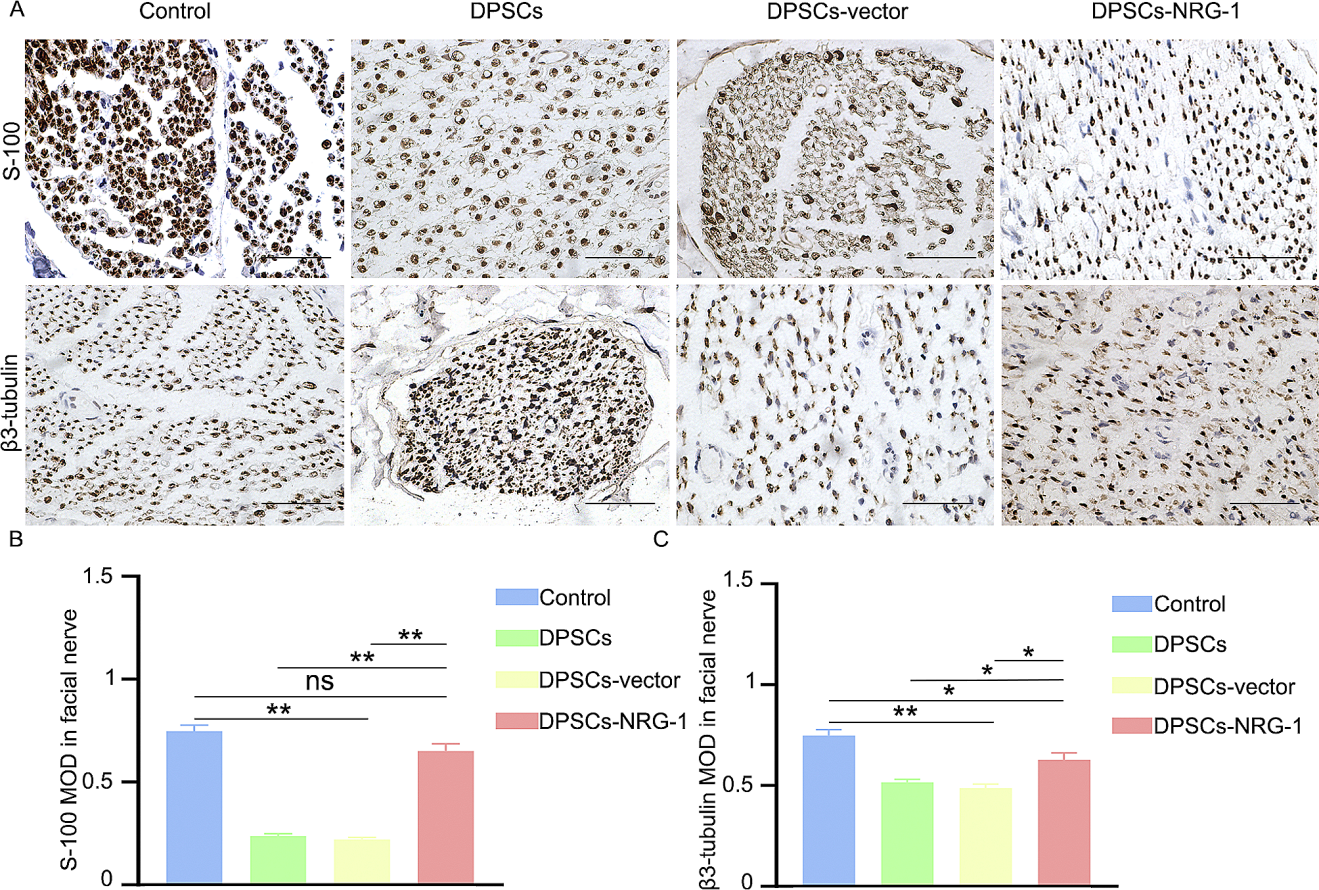
NRG-1 promotes repair of facial nerve injury. (**A**) The immunohistochemical technique was employed to ascertain the expression of axon and myelin markers, namely S-100 and β3-tubulin. (**B**, **C**) The average optical density obtained from immunohistochemistry was subjected to statistical analysis. (ns (*P* > 0.05), * (*P* < 0.05), ** (*P* < 0.001))

At the 8-week mark, IHC analysis indicated significantly higher levels of axonal and myelin markers S-100 and β3-tubulin in the DPSCs-NRG-1 group compared to other injury groups. However, no significant differences were observed between the DPSCs group and the DPSCs-vector group (Fig. 6 (A, B, C)). Supplementary Figs. 3 and 4 show expression at 2 and 4 weeks, respectively. Moreover, S-100 and β3-tubulin expression detected by RT-PCR corroborated the histochemistry results (Supplementary Fig. 5).

**Fig. 7:**
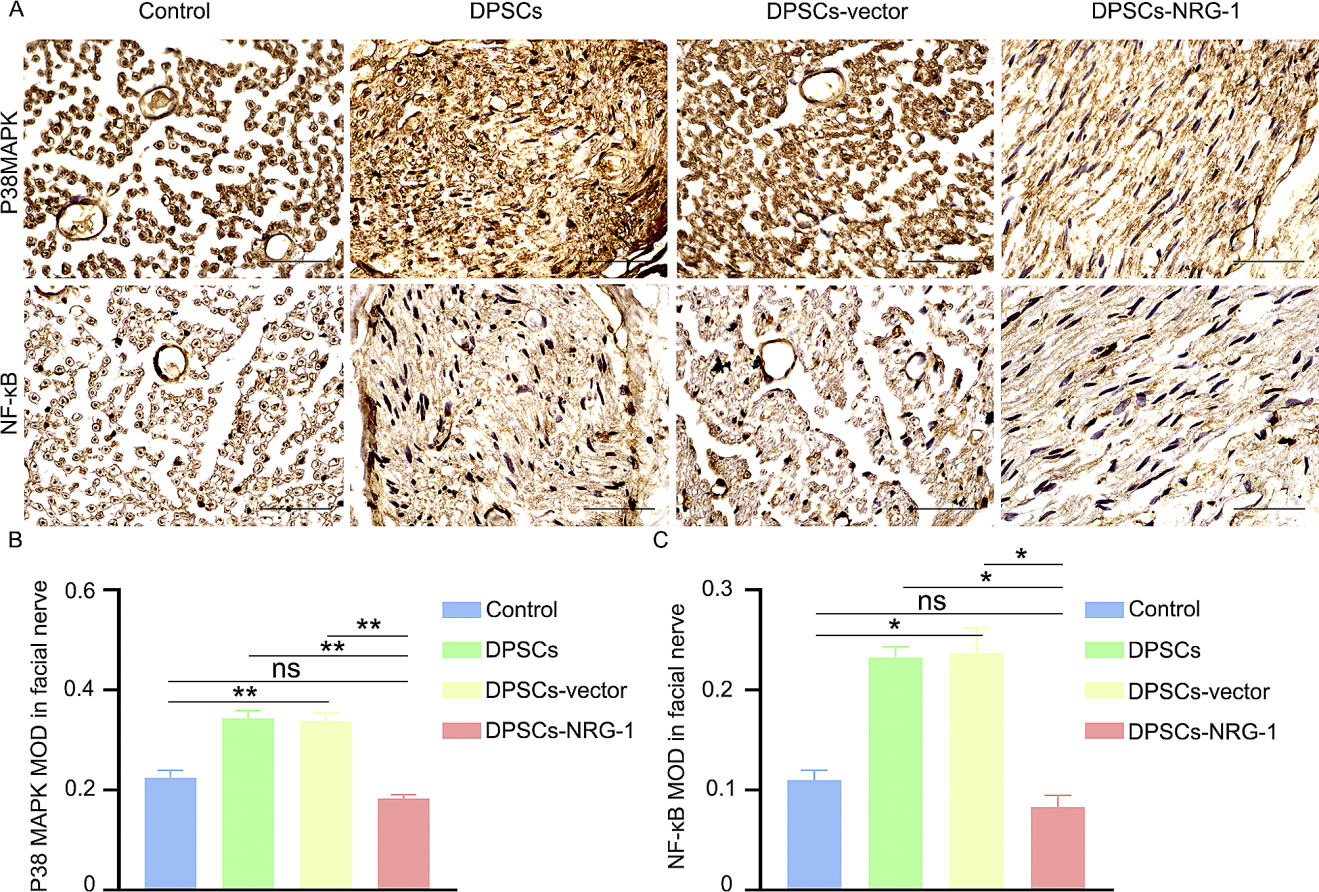
NRG-1 is known to inhibit P38 MAPK, NF-κB. (**A**) The immunohistochemical technique was employed to ascertain the expression of P38 MAPK and NF-κB. (**B**, **C**) The average optical density obtained from immunohistochemistry was subjected to statistical analysis. (ns (*P* > 0.05), * (*P* < 0.05), ** (*P* < 0.001))

Additionally, an evaluation of P38 MAPK and NF-κB expression levels in each experimental group was conducted. The IHC results revealed a notable reduction in P38 MAPK and NF-κB signaling pathway expression in the DPSCs-NRG-1 group compared to other injury groups (Fig. 7(A, B, C)).

These findings suggest that the morphology of organ tissues remained unchanged (Supplementary Fig. 6).

## Discussion

Addressing facial nerve injury remains a challenge for clinicians, despite advancements in therapeutic drugs, which have not sufficiently resolved this issue. Recently, DPSCs have gained attention for their potential in nerve injury repair, yet their effectiveness has been limited, necessitating additional gene integration to realize their full potential. This study focused on NRG-1 expression and function in DPSCs, demonstrating its role in enhancing proliferation, migration, neurotropic differentiation, and cytoskeletal rearrangement in DPSCs. Moreover, NRG-1 contributed to the in vivo repair process of facial nerve injury by DPSCs, suggesting its potential in facilitating recovery.

DPSCs are noted for their extensive multidirectional differentiation potential among various mesenchymal stem cell types [20], coupled with their ease of acquisition, low immunogenicity, and responsiveness to diverse cytokines. These characteristics enable DPSCs to differentiate into neurons or neuron-like cells, making them promising candidates in tissue engineering for neurological disorder treatments and tissue restoration [21]. Consequently, rat dental pulp stem cells were chosen for this research. However, challenges such as the lack of specific identifiers for DPSCs, inherent variability of MSCs, and limitations of traditional two-dimensional culture methods, which impact long-term pluripotency maintenance, restrict their application in tissue engineering. Identifying target genes that can enhance DPSC functionality is therefore crucial for enabling their effective application.

NRG-1, a member of the EGF family [14], is known to promote the proliferation, migration, and differentiation of various cell types [22]. However, its specific role in DPSCs has been unclear. This study shows that NRG-1 enhances DPSC proliferation and migration, but the underlying mechanisms require further exploration. In our study, RT-PCR analysis assessed astrocyte markers S-100 and GFAP, and neuronal markers β3-tubulin and NSE. The results indicated increased expression of neuronal markers β3-tubulin and NSE in the Ne-OV group, with no significant impact on astrocyte markers S-100 and GFAP. This suggests that NRG-1 may promote DPSC differentiation into neuron-like cells.

The role of RhoA in nerve regeneration is complex. Its activation in neurons hinders axon regeneration [23], while in astrocytes, it facilitates regeneration [24]. In neurons, RhoA blocks axon regeneration by promoting actin polymerization and reducing growth cone size and dynamics, impeding neural growth. This study observed a downregulation of RhoA in the Ne-OV group. Phalloidin staining showed reduced microfilament actin polymerization in this group. These findings imply that NRG-1 might enhance DPSC differentiation into neuron-like cells through the RhoA/F-actin pathway, a mechanism requiring further investigation.

Previous studies have demonstrated the inhibitory effects of NRG-1 on fibrosis [25], as well as its capacity to resist cellular senescence [21] and apoptosis [26]. Moreover, NRG-1 has been observed to mitigate mitochondrial dysfunction [22], stimulate cell proliferation and migration [27], induce differentiation [28, 29], promote angiogenesis [22], and exhibit neuroprotective properties.

Therefore, our study aimed to assess the effect of NRG-1 on in vivo facial nerve injury restoration in rats. Following successful injury induction, samples were collected at 2, 4, and 8 weeks post-injury to evaluate facial nerve repair progress. At the 8-week mark, we observed restoration of action potential and reflex waves in the facial nerve trunk of rats in the DPSCs-NRG-1 group.

Analysis of Hematoxylin and Eosin (H&E) staining and Transmission Electron Microscopy (TEM) revealed that myelin sheath thickness and axon count were significantly higher in the DPSCs-NRG-1 group compared to other injury groups. IHC was used to assess axon and myelin markers, specifically β3-tubulin and S-100. At 2 weeks, no significant differences were noted among the injury groups. However, at 4 and 8 weeks, the DPSCs-NRG-1 group showed notably higher levels of β3-tubulin and S-100 compared to the DPSCs and DPSCs-vector groups. These results suggest that NRG-1 plays a role in facilitating recovery from facial nerve injury, although the exact mechanism remains to be investigated.

After peripheral nerve injury, an initial inflammatory reaction occurs, aiming to remove deteriorated axons and dismantle the myelin sheath, thereby promoting axon regeneration [30]. Schwann cells rapidly activate NF-κB and P38 MAPK, releasing various pro-inflammatory factors in response to injury stress, which collectively aid in recruiting and infiltrating blood-derived macrophages around the damaged nerves.

The process outlined above initiates a feedback mechanism that promotes differentiation and clustering of Schwann cells, encourages formation of the Bonner zone, and aids in the elongation of the growth cone, while also impeding axon regeneration. Previous research has shown a significant link between P38 MAPK and hippocampal neuron apoptosis [31]. Immunohistochemistry was used to measure NF-κB and P38 MAPK expression in rats with facial nerve injury treated with DPSCs expressing high levels of NRG-1.

Our results revealed a decrease in NF-κB and P38 MAPK expression in the facial nerve tissue of the DPSCs-NRG-1 group. This suggests that NRG-1 might inhibit NF-κB and P38 MAPK signaling pathways, resulting in reduced expression. Activation of the MAPK signaling pathway is known to reduce inflammatory cascade amplification, enhance cell proliferation and axon regeneration, and aid in facial nerve injury repair.

While NRG-1’s role in promoting DPSC recruitment is recognized, the exact number of cells reaching the facial nerve injury site is unclear. As an alternative, incorporating NRG-1 into a biocompatible scaffold is suggested to better accommodate DPSCs. This method aims to increase cell recruitment at the injury site by reducing the inflammatory cascade, thus promoting cell proliferation and axon regeneration, and ultimately aiding facial nerve injury repair.

## Conclusion

NRG-1 possesses the ability to facilitate the repair of facial nerve injuries by promoting the regenerative capacity of DPSCs. Therefore, NRG-1 holds significant potential as a pivotal gene involved in the reparative processes of nerve damage.

### Electronic supplementary material

Below is the link to the electronic supplementary material.

Supplementary Material 1

Supplementary Material 2

Supplementary Material 3

Supplementary Material 4

Supplementary Material 5

Supplementary Material 6

Supplementary Material 7

Supplementary Material 8

Supplementary Material 9

Supplementary Material 10

## Data Availability

No datasets were generated or analysed during the current study.

## Abbreviations

ANOVA: A one-way analysis of variance
bFGF: Basic Fibroblast Growth Factor
BDNF: Brain-Derived Neurotrophic Factor
B27: HLA-27
CCK-8: Cell Counting Kit-8
CMAP: Compound Muscle Action Potential
DPSCs: Dental Pulp Stem Cells
EGF: Epidermal Growth Factor
F-actin: Cytoskeletal actin fibers
GDNF: Glial Cell Line-Derived Neurotrophic Factor
GFAP: Glial Fibrillary Acidic Protein
H&E Staining: Hematoxylin-eosin Staining
IF: Immunoflurescence
IHC: Immunohistochemistry
MSCs: Mesenchymal Stem Cells
NT3: Neurotrophin-3
NRG-1: Neureglin-1
NSE: Neuron Specific Enolase
NMJ: Neuromuscular Junction
PFA: Paraformaldehyde
PBS-T: Phosphate-Buffered Saline with Tween-20
PBS-B: Phosphate-Buffered Saline with Bovine Serum Albumin
RT-PCR: Reverse Transcription Polymerase Chain Reaction
RhoA: Ras homologous gene family member A
S-100: Calcium-binding protein
TEM: Transmission Electron Microscope
