# Supplementary figures and images for: The involvement of Neuregulin-1 in the process of facial nerve injury repair through the utilization of dental pulp stem cells
Source: BMC Oral Health. 2024 Feb 14;24:238. doi: 10.1186/s12903-024-03953-z (PMC10868091; doi:10.1186/s12903-024-03953-z)

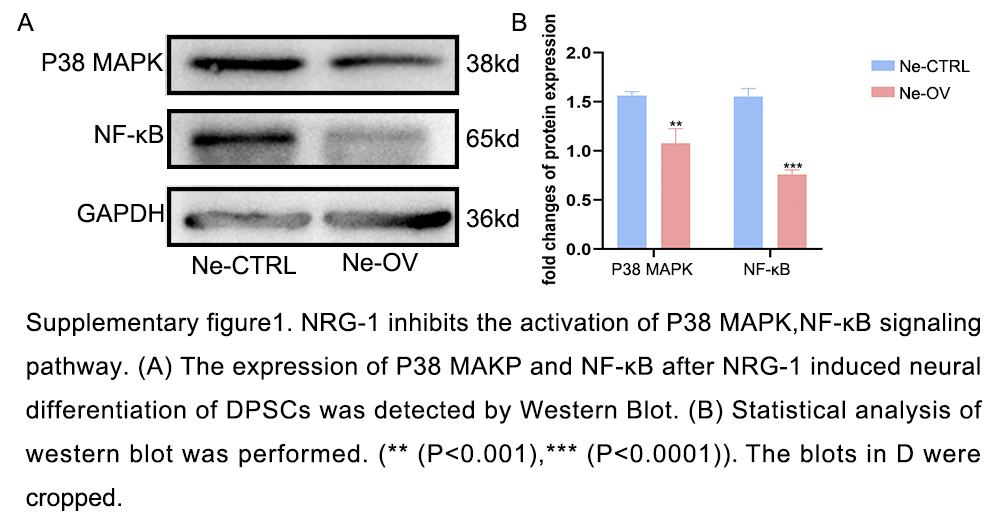

Supplement: Supplementary file 1 — Supplementary Material 1 [file 12903_2024_3953_MOESM1_ESM.tif]

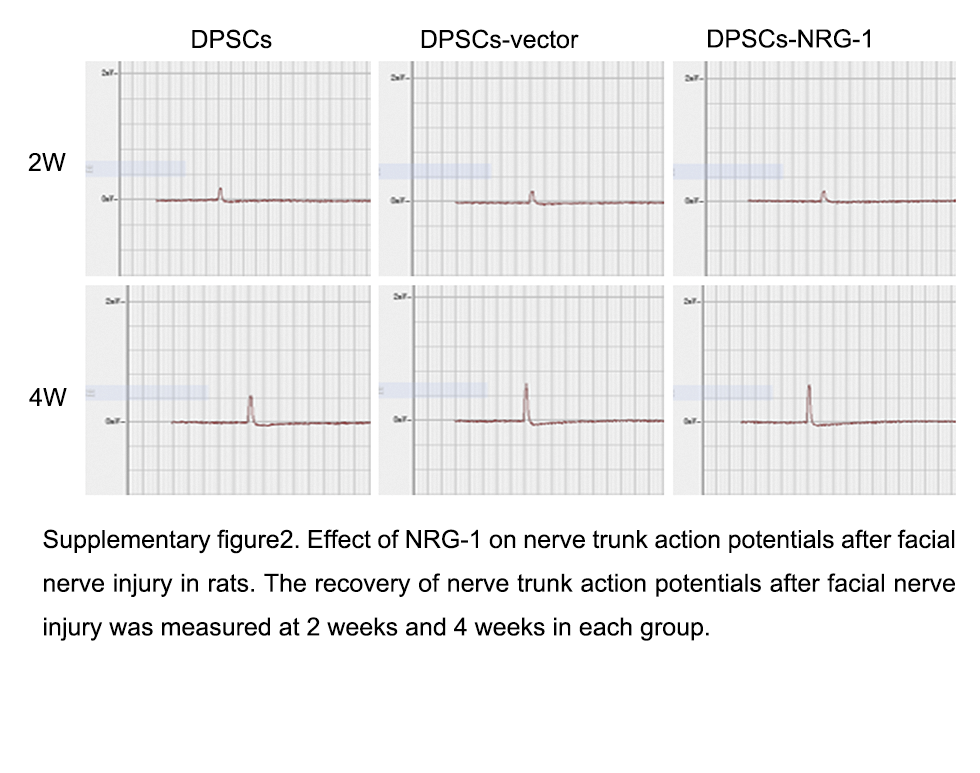

Supplement: Supplementary file 2 — Supplementary Material 2 [file 12903_2024_3953_MOESM2_ESM.tif]

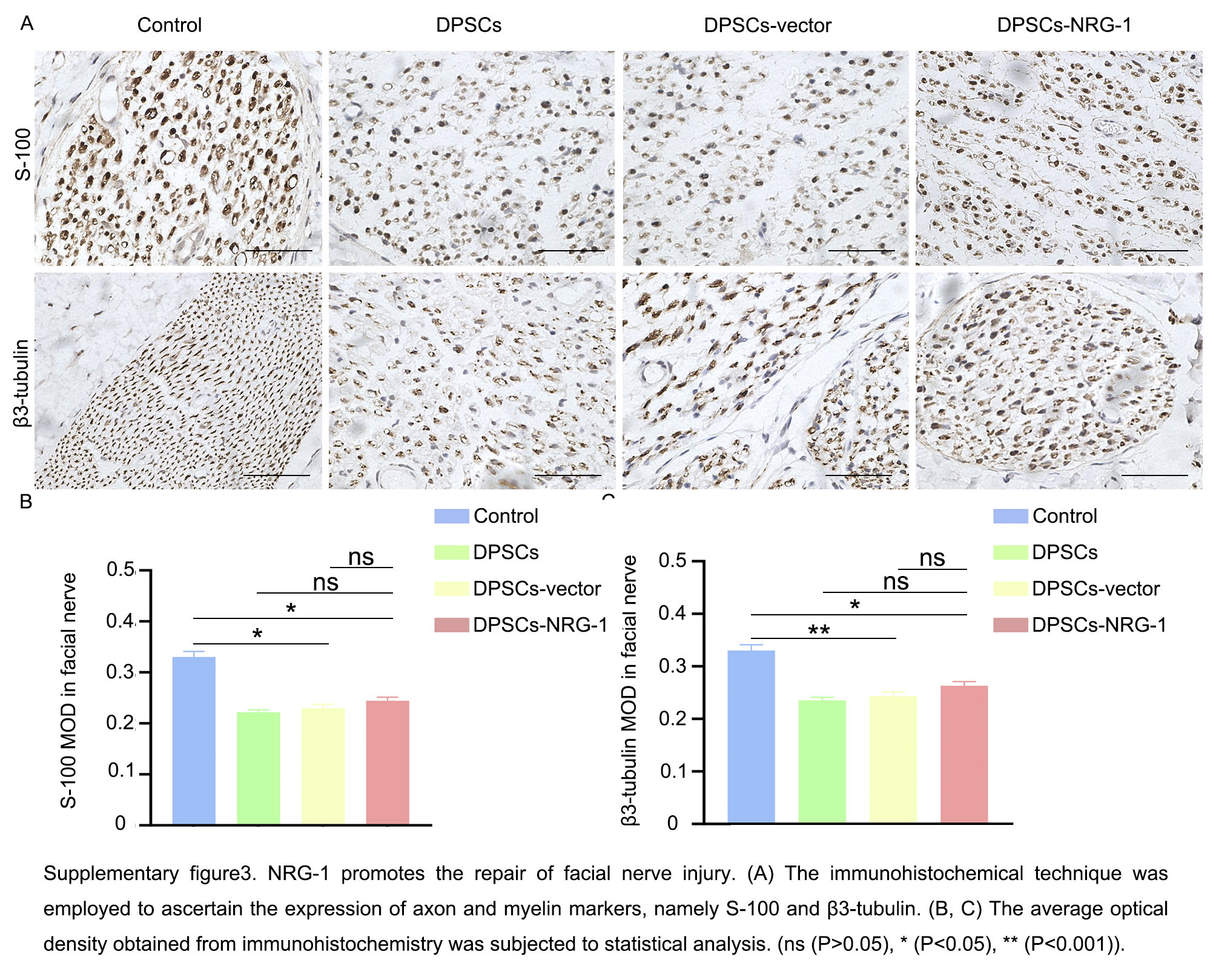

Supplement: Supplementary file 3 — Supplementary Material 3 [file 12903_2024_3953_MOESM3_ESM.tif]

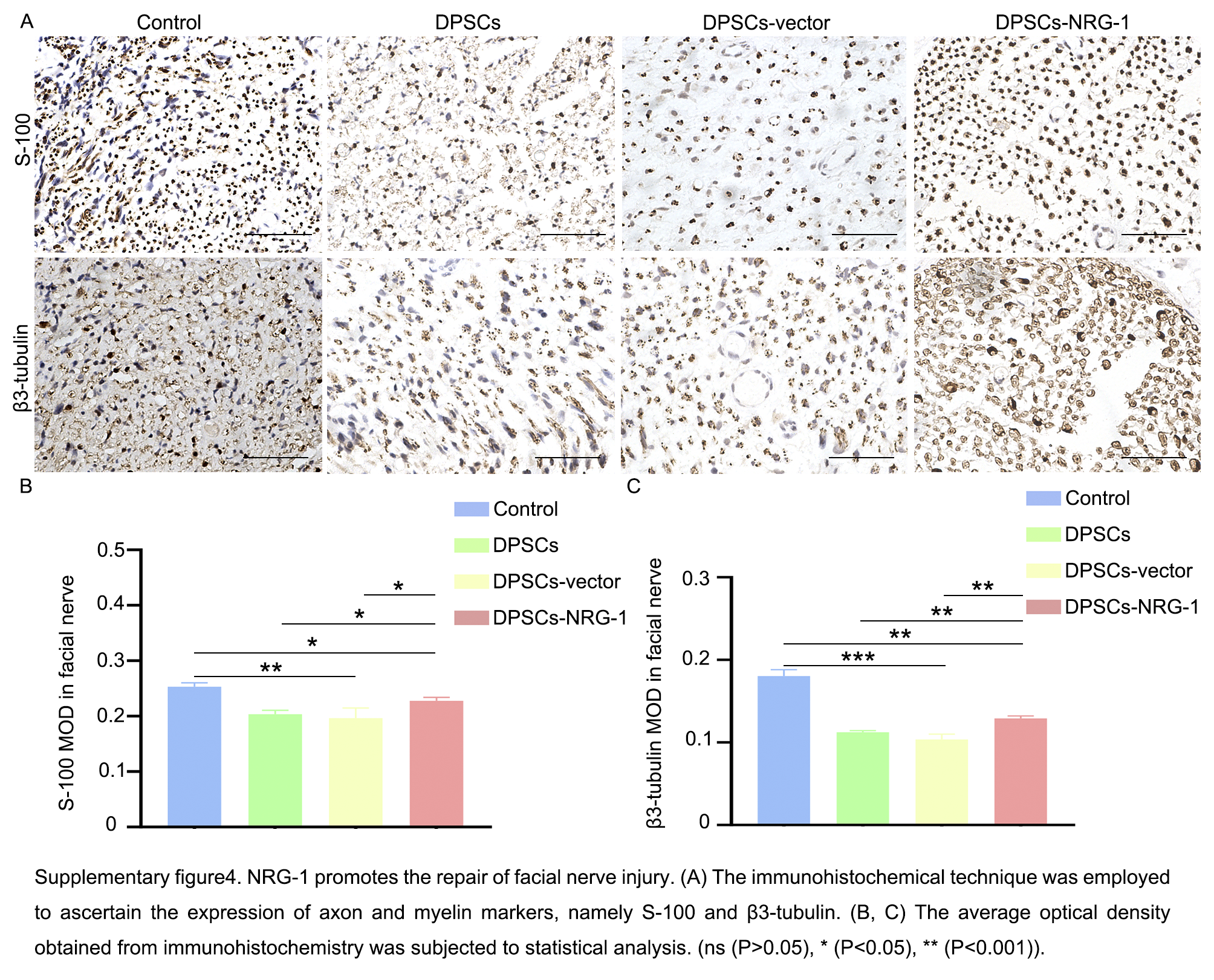

Supplement: Supplementary file 4 — Supplementary Material 4 [file 12903_2024_3953_MOESM4_ESM.tif]

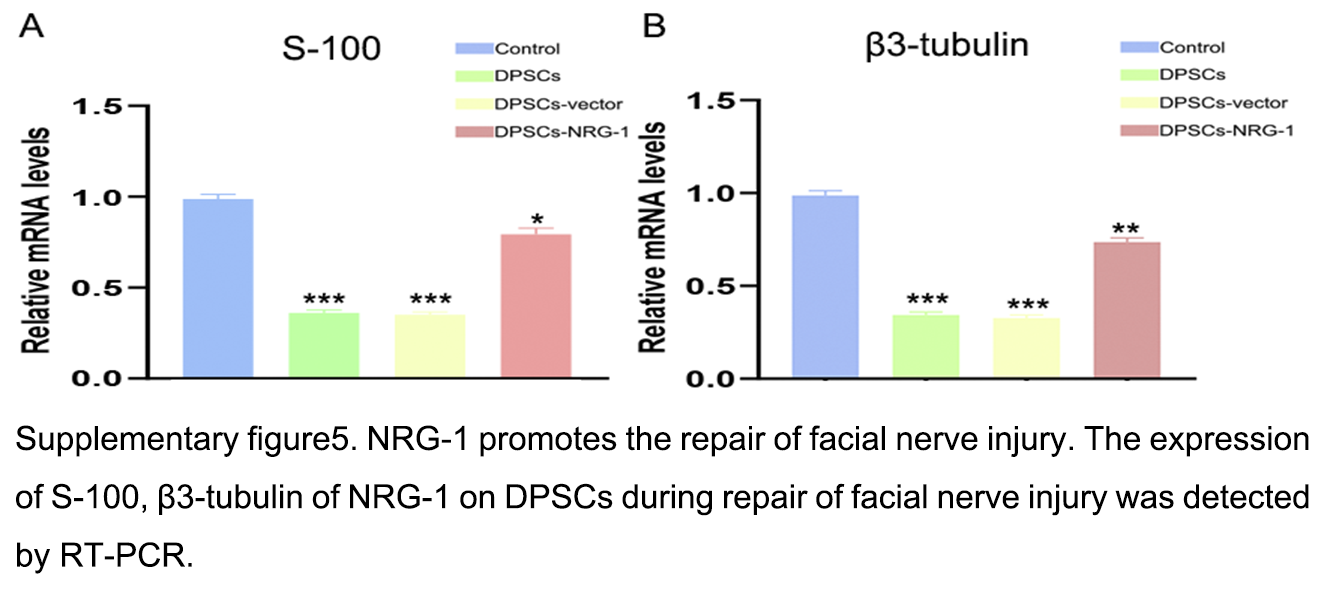

Supplement: Supplementary file 5 — Supplementary Material 5 [file 12903_2024_3953_MOESM5_ESM.tif]

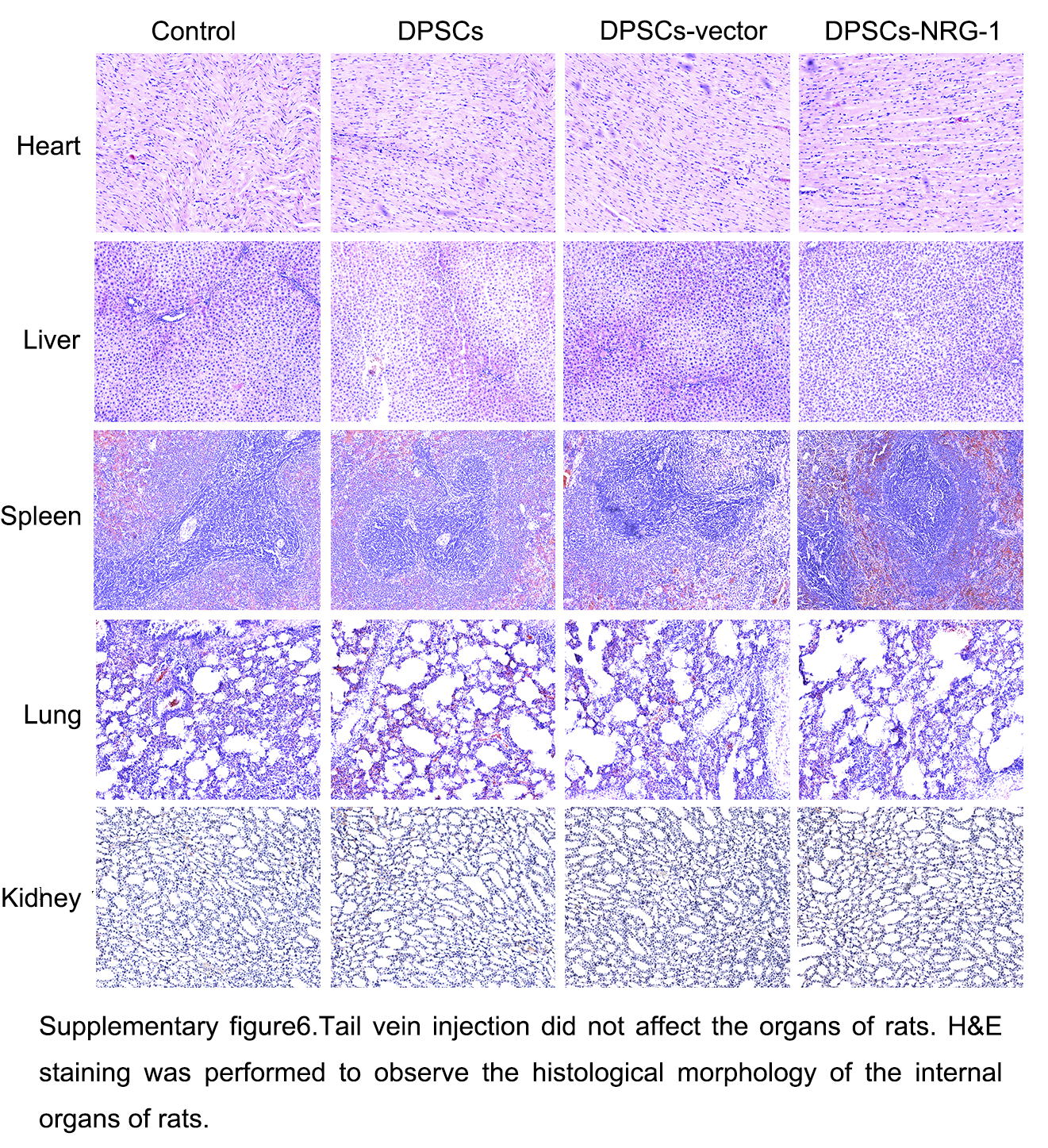

Supplement: Supplementary file 6 — Supplementary Material 6 [file 12903_2024_3953_MOESM6_ESM.tif]
