# Supplementary material for: The involvement of Neuregulin-1 in the process of facial nerve injury repair through the utilization of dental pulp stem cells
Source: BMC Oral Health. 2024 Feb 14;24:238. doi: 10.1186/s12903-024-03953-z (PMC10868091; doi:10.1186/s12903-024-03953-z)
Supplement: Supplementary file 7 — Supplementary Material 7 [file 12903_2024_3953_MOESM7_ESM.pdf]

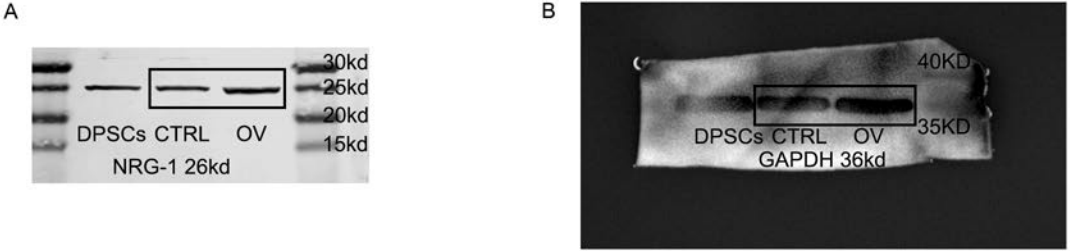

A. As shown in Figure 1B, Western Blot analysis of NRG-1 was presented in the black rectangle of A. The DPSCs group was on the left, the CTRL group transfected with lentivirus without target gene was in the middle, and the OV sample transfected with target gene NRG-1 was shown on the right. The molecular weight of the NRG-1 protein was determined to be 26kd and it also contained a protein marker on the right side of the image. It is worth noting that the bands corresponding to the 15kd, 20kd, 25kd and 35kd labels are retained in the figure. Notably, the image has not been extensively processed including high contrast adjustments or multiple exposures, and the main information is appropriately labeled in the image.B. Western Blot analysis of GAPDH as shown in Figure 1B is shown in the black rectangle of b, with DPSCs group at the far left, CTRL group transfected with lentivirus without target gene in the middle, and OV sample transfected with target gene NRG-1 shown on the right. The molecular weight of GAPDH protein was determined to be 36kd, and a protein marker was also included on the left side of the image. It is worth noting that the bands corresponding to the 35kd and 40kd labels are retained in the figure. Notably, the image has not been extensively processed including high contrast adjustments or multiple exposures, and the main information is appropriately labeled in the image.
