# Supplementary material for: The involvement of Neuregulin-1 in the process of facial nerve injury repair through the utilization of dental pulp stem cells
Source: BMC Oral Health. 2024 Feb 14;24:238. doi: 10.1186/s12903-024-03953-z (PMC10868091; doi:10.1186/s12903-024-03953-z)
Supplement: Supplementary file 8 — Supplementary Material 8 [file 12903_2024_3953_MOESM8_ESM.pdf]

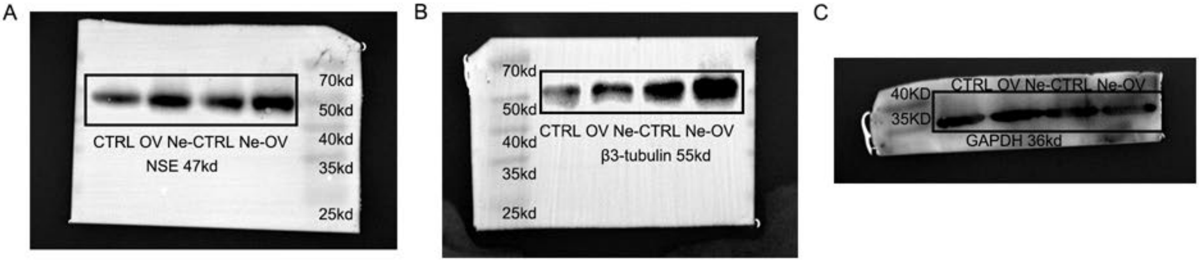

A.The Western Blot analysis of NSE depicted in Figure 2D is presented within the confines of a black rectangle of A. The molecular weight of the NSE protein is determined to be 47kd, and a protein marker is also included on the right side of the image. Notably, the figure retains bands corresponding to markers of 25kd, 35kd, 40kd, 50kd and 70kd. It is important to note that the image has not undergone extensive processing involving high contrast adjustments or multiple exposures, and the primary information is duly marked within the image.

B.The Western Blot analysis of β3-tubulin depicted in Figure 2D is presented within the confines of a black rectangle of B. The molecular weight of the β3-tubulin protein is determined to be 55kd, and a protein marker is also included on the left side of the image. Notably, the figure retains bands corresponding to markers of 25kd,35kd, 40kd, 50kd and 70kd. It is important to note that the image has not undergone extensive processing involving high contrast adjustments or multiple exposures, and the primary information is duly marked within the image.

C.The Western Blot analysis of GAPDH depicted in Figure 2D is presented within the confines of a black rectangle of C. The molecular weight of the GAPDH protein is determined to be 36kd, and a protein marker is also included on the left side of the image. Notably, the figure retains bands corresponding to markers of 35kd, 40kd. It is important to note that the image has not undergone extensive processing involving high contrast adjustments or multiple exposures, and the primary information is duly marked within the image.
