# Supplementary material for: The involvement of Neuregulin-1 in the process of facial nerve injury repair through the utilization of dental pulp stem cells
Source: BMC Oral Health. 2024 Feb 14;24:238. doi: 10.1186/s12903-024-03953-z (PMC10868091; doi:10.1186/s12903-024-03953-z)
Supplement: Supplementary file 9 — Supplementary Material 9 [file 12903_2024_3953_MOESM9_ESM.pdf]

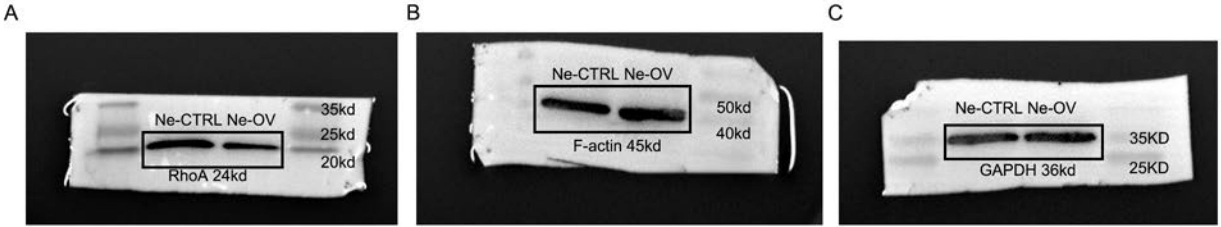

A.The Western Blot analysis of RhoA depicted in Figure 3B is presented within the confines of a black rectangle of A. The molecular weight of the F-actin protein is determined to be 24kd, and a protein marker is also included on the right side of the image. Notably, the figure retains bands corresponding to markers of 20kd, 25kd, 35kd . It is important to note that the image has not undergone extensive processing involving high contrast adjustments or multiple exposures, and the primary information is duly marked within the image.

B.The Western Blot analysis of F-actin depicted in Figure 3B is presented within the confines of a black rectangle of B. Notably, the figure retains bands corresponding to markers of 40kd, 50kd . It is important to note that the image has not undergone extensive processing involving high contrast adjustments or multiple exposures, and the primary information is duly marked within the image.

C.The Western Blot analysis of GAPDH depicted in Figure 3B is presented within the confines of a black rectangle of B. The molecular weight of the GAPDH protein is determined to be 36kd, and a protein marker is also included on the left side of the image. Notably, the figure retains bands corresponding to markers of 25kd, 35kd. It is important to note that the image has not undergone extensive processing involving high contrast adjustments or multiple exposures, and the primary information is duly marked within the image.
