# Supplementary material for: The involvement of Neuregulin-1 in the process of facial nerve injury repair through the utilization of dental pulp stem cells
Source: BMC Oral Health. 2024 Feb 14;24:238. doi: 10.1186/s12903-024-03953-z (PMC10868091; doi:10.1186/s12903-024-03953-z)
Supplement: Supplementary file 10 — Supplementary Material 10 [file 12903_2024_3953_MOESM10_ESM.pdf]

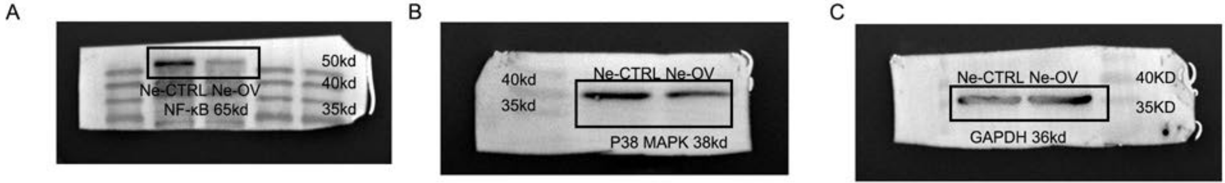

A.The Western Blot analysis of NF-κB depicted in Supplement Figure 1A is presented within the confines of a black rectangle of A. The molecular weight of the NF-κB protein is determined to be 65kd, and a protein marker is also included on the right side of the image. Notably, the figure retains bands corresponding to markers of 35kd, 40kd, 50kd . It is important to note that the image has not undergone extensive processing involving high contrast adjustments or multiple exposures, and the primary information is duly marked within the image.

B.The Western Blot analysis of P38 MAPK depicted in Supplement Figure 1A is presented within the confines of a black rectangle of B. Notably, the figure retains bands corresponding to markers of 35kd, 40kd . It is important to note that the image has not undergone extensive processing involving high contrast adjustments or multiple exposures, and the primary information is duly marked within the image.

C.The Western Blot analysis of GAPDH depicted in Supplement Figure 1A is presented within the confines of a black rectangle of B. The molecular weight of the GAPDH protein is determined to be 36kd, and a protein marker is also included on the left side of the image. Notably, the figure retains bands corresponding to markers of 35kd, 40kd. It is important to note that the image has not undergone extensive processing involving high contrast adjustments or multiple exposures, and the primary information is duly marked within the image.
